# Genome engineering of mammalian haploid embryonic stem cells using the Cas9/RNA system

**DOI:** 10.7717/peerj.230

**Published:** 2013-12-23

**Authors:** Takuro Horii, Sumiyo Morita, Mika Kimura, Ryouhei Kobayashi, Daiki Tamura, Ryou-u Takahashi, Hironobu Kimura, Isao Suetake, Hirokazu Ohata, Koji Okamoto, Shoji Tajima, Takahiro Ochiya, Yumiko Abe, Izuho Hatada

**Affiliations:** 1 Laboratory of Genome Science, Biosignal Genome Resource Center, Institute for Molecular and Cellular Regulation, Gunma University, Maebashi, Gunma, Japan; 2 Department of Laboratory Sciences, Graduate School of Health Sciences, Gunma University, Maebashi, Gunma, Japan; 3 Division of Molecular and Cellular Medicine, National Cancer Center Research Institute, Chuo-ku, Tokyo, Japan; 4 Laboratory of Epigenetics, Institute for Protein Research, Osaka University, Suita, Osaka, Japan; 5 Division of Cancer Development System, National Cancer Center Research Institute, Chuo-ku, Tokyo, Japan

**Keywords:** CRISPR/Cas, Haploid, Embryonic stem cells, Genome engineering, *Tet1*, *Tet2*, *Tet3*

## Abstract

Haploid embryonic stem cells (ESCs) are useful for studying mammalian genes because disruption of only one allele can cause loss-of-function phenotypes. Here, we report the use of haploid ESCs and the CRISPR RNA-guided Cas9 nuclease gene-targeting system to manipulate mammalian genes. Co-transfection of haploid ESCs with vectors expressing Cas9 nuclease and single-guide RNAs (sgRNAs) targeting *Tet1*, *Tet2*, and *Tet3* resulted in the complete disruption of all three genes and caused a loss-of-function phenotype with high efficiency (50%). Co-transfection of cells with vectors expressing Cas9 and sgRNAs targeting two loci on the same chromosome resulted in the creation of a large chromosomal deletion and a large inversion. Thus, the use of the CRISPR system in combination with haploid ESCs provides a powerful platform to manipulate the mammalian genome.

## Introduction

Generation of homozygous mutant mammalian cells is complicated because they have a diploid genome. If one allele of an autosomal gene is disrupted, the resulting heterozygous mutant may not display a phenotype due to the existence of the other allele; therefore, studying the functions of genes in mammalian cells can be challenging. The “complication” of ES cell diploidy for genetic analysis has been addressed either by selecting “targeted” clones undergoing LOH (facilitated by mutagenesis using a hypomorphic neor gene) or by sequential targeting both chromosomes using different resistance genes (Mortensen et al., 1991; Milstone, Bradwin & Mortensen, 1999). Haploid cells contain only one copy of each chromosome and disruption of one allele can directly cause loss-of-function phenotypes. Recently, mouse haploid embryonic stem cells (ESCs) have been successfully generated, providing an ideal tool for genetic analyses (Elling et al., 2011; Leeb & Wutz, 2011). Haploid ESCs retain the majority of the biological aspects of normal diploid ESCs, except for their unusual karyotype. A genome-wide expression analysis revealed that the expression profiles of haploid and diploid ESCs, including stem cell markers, are almost identical (Leeb & Wutz, 2011). Haploid ESCs are able to differentiate into a wide range of cell types both *in vitro* and in chimeric embryos produced by blastocyst injection. During differentiation, the cells gain a diploid karyotype (Leeb & Wutz, 2011). Remarkably, haploid ESCs are germline competent in chimeric mice (Leeb et al., 2012; Li et al., 2012; Yang et al., 2012).

The recent development of site-specific endonucleases for selective genome cleavage has been an important advancement in mammalian genome engineering. These enzymes include zinc-finger nucleases (Porteus & Carroll, 2005), transcription activator-like effector nucleases (Miller et al., 2011), and clustered regularly interspaced short palindromic repeats (CRISPR) RNA-guided Cas9 nucleases (Cong et al., 2013; Mali et al., 2013). Zinc-finger nucleases and transcription activator-like effector nucleases are composed of programmable, sequence-specific DNA-binding modules linked to a non-specific DNA cleavage domain. CRISPR RNA-guided Cas9 nucleases use small base-pairing RNAs to target and cleave foreign DNA elements in a sequence-specific manner (Wiedenheft, Sternberg & Doudna, 2012). Among these technologies, the type II CRISPR system from *Streptococcus pyogenes* is the simplest. In this system, a single gene encoding the Cas9 protein and two RNAs, a mature CRISPR RNA (crRNA) and a partially complementary trans-acting RNA (tracrRNA), are sufficient for RNA-guided cleavage of foreign DNAs (Jinek et al., 2012). Maturation of crRNA requires RNase III and tracrRNA (Deltcheva et al., 2011); however, this process can be bypassed by using an engineered small guide RNA (sgRNA) containing a hairpin that mimics the tracrRNA-crRNA complex and a short sequence complementary to the target DNA (Jinek et al., 2012). The Cas9 endonuclease can generate sequence-specific double-strand breaks of target DNAs bound to sgRNAs. The binding site of a target DNA requires a protospacer-adjacent motif (PAM) (with the sequence NGG) juxtaposed to the DNA complementary region (Marraffini & Sontheimer, 2010). Therefore, the CRISPR RNA-guided Cas9 nuclease system requires only two molecules: the Cas9 protein and a sgRNA for host-independent gene-targeting.

Here, we describe a new platform for simple genetic manipulation of the mammalian genome that uses a combination of the CRISPR RNA-guided Cas9 nuclease system and haploid ESCs.

## Materials and Methods

### Parthenogenetic activation

Oocytes were collected from superovulated B6DBAF1 and B6-EGFP females and were activated in calcium free M16 medium containing 5 mM strontium chloride. After activation for 3 h, the embryos were subsequently cultured in M16 medium. All animal experiments were approved by the Animal Care and Experimentation Committee of Gunma University, Showa Campus, Japan.

### Generation of haploid ES cell lines

ESC derivation was performed as described previously with minor modifications (Leeb & Wutz, 2011; Horii et al., 2008). Briefly, the zonas of morula stage embryos were removed and then cultured in chemically defined ES medium supplemented with 3 µM CHIR99021 and 1 µM PD0325901 (2i).

### Fluorescence-activated cell sorting (FACS) analysis

The cells were stained with 15 µg/ml Hoechst 33342 (Invitrogen) and then cell sorting for DNA content was performed using a FACS Aria III cell sorter (Becton Dickinson). The haploid 1n peak was purified. The cells were fixed in 70% ethanol, digested with RNase and stained with propidium iodide, and then analytic flow profiles of DNA content were recorded using a FACS Calibur flow cytometer (Becton Dickinson).

### Transfection of cells

The generated haploid ES cell lines were cultured on gelatine-coated plates under standard ESC culture conditions. Before transfection ESCs were sorted and haploid fractions were collected (Fig. S1). The cells were co-transfected with a plasmid expressing mammalian codon-optimized Cas9 under the control of a CAG promoter and plasmids expressing sgRNAs under the control of a U6 promoter (Horii et al., 2013), along with a linear puromycin marker (Clontech). Transfections were performed using Lipofectamine 2000 reagent (Life Technologies) according to the manufacturer’s instructions. In the triple targeting experiments, cells were co-transfected with a plasmid expressing mammalian codon-optimized Cas9 and three plasmids expressing sgRNAs targeting *Tet1*, *Tet2* and *Tet3*, along with a linear puromycin marker (Clontech). Twelve hours after transfection, ESCs were replated at a low density. One day after replating, cells were incubated with 1 µg/ml puromycin for 48 h. After recovering for 4 to 6 days, individual colonies were picked and genotyped by PCR-RFLP, or passaged several times and frozen. Full details of the targeted sequences and primers are shown in Table S2.

### Assay for genome modification

To detect small genome modifications, PCRs were performed using primers flanking the targeted regions (Table S2). The PCR products were digested with BfuAI or MnII, which cleave at the Cas9 target site of non-modified genomes, and then analysed by to gel electrophoresis. To detect large deletions and inversions, PCRs were performed using primers flanking the target sites of the two *Tet1*-specific sgRNAs (Table S2, Figs. 4A and 4B). A deletion was indicated by the production of a ∼300 bp product. An inversion was indicated by the production of ∼190 bp and ∼260 bp products. All of the mutant PCR products were cloned into a TA-cloning vector (pCR2.1) and the mutations were confirmed by DNA sequencing.

### Quantification of the global 5-hydroxymethylcytosine (5hmC) content

The determination of the 5hmC levels in DNA from triple-targeted haploid ESC clones was performed as described previously with slight modifications (Szwagierczak et al., 2010). Briefly, 200 ng of genomic DNA was incubated with 10 pmol of β-GT and 1.91 kBq of [3H]-UDP-glucose (Perkin Elmer) at 25°C in a 25 µl reaction buffer comprising 50 mM potassium acetate, 10 mM magnesium acetate, 1 mM DTT and 20 mM Tris-acetate (pH 7.9). After 1 h, the mixture was added to 20 µg of proteinase K in 1% (w/v) SDS and then incubated at 55°C for 30 min. After the incubation, the reaction mixture was spotted onto a DE81 filter disc (GE Healthcare). The disc was washed as described previously (Suetake et al., 2003), and the incorporated radioactivity was determined using an LS-5000 scintillation counter (Beckman).

### Quantification of the 5hmC content of *Ecat1*

The 5hmC content of the *Ecat1* gene was measured using the Quest 5-hmC Detection Kit (Zymo Research, Irvine). This kit enables sequence-specific detection of 5hmC within DNA; utilizing a 5hmC glucosyltransferase, 5hmC in DNA is specifically tagged with a glucose moiety yielding the modified base glucosyl-5hmC. After glucosylation of 5hmC, the DNA was digested with a glucosyl-5hmC-sensitive restriction endonuclease (MspI) and then quantitative PCR was performed using *Ecat1*-specific primers: 5′-GGAGAGCACATCCCACATCT-3′ and 5′-GTGAGCCAGATCAGTGAGCA-3′.

## Results

### Generation of haploid ESCs from mouse embryos

To generate haploid mouse embryos, unfertilized oocytes isolated from superovulated B6DBAF1 hybrid female mice and B6-EGFP mice were activated using strontium chloride in the calcium free M16 medium. After culture in M16 medium, 26 morulae were obtained from 58 activated B6DBAF1 oocytes and were used for generation of ESCs (Table S1). Inner cell masses were cultured in chemically defined dual inhibition (2i) ES medium supplemented with 3 µM CHIR99021 and 1 µM PD0325901 to inhibit glycogen synthase kinase 3 and mitogen-activated protein kinase, respectively. A total of 17 B6DBAF1 ESC lines were obtained, of which 82% had haploid DNA content (Table S1). Similar results were also obtained for B6-EGFP mice (Table S1); in this inbred strain, 70% of the 10 ESC lines obtained had haploid DNA content.

### Targeting single genes in haploid ESCs

**Figure 1:**
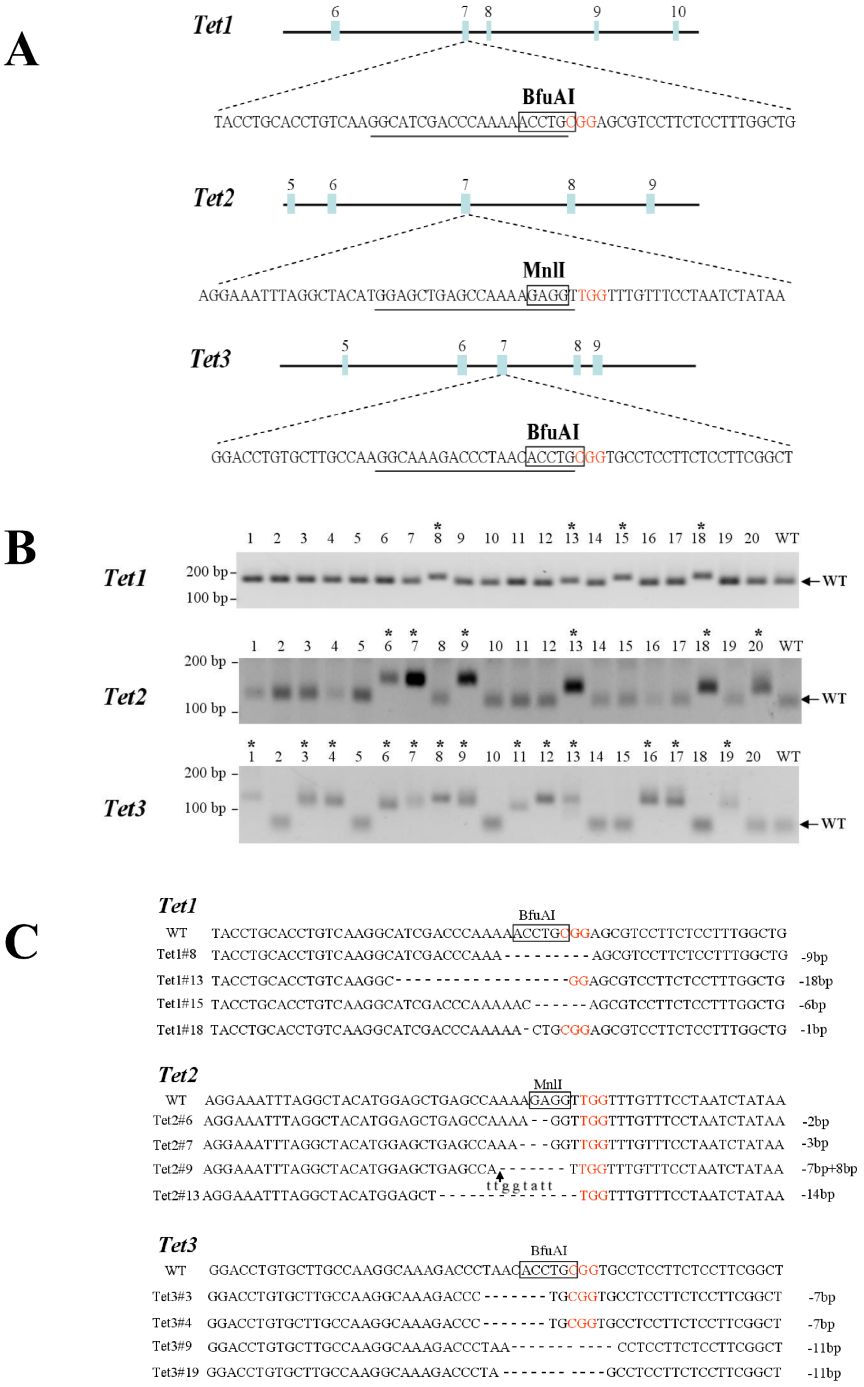
Identification and sequencing of successfully targeted *Tet1, Tet2*, and *Tet3* genes in mouse haploid ESCs. (A) The Cas9/sgRNA-targeting sites in mouse *Tet1*, *Tet2* and *Tet3*. The sgRNA-targeting sequences are underlined and the PAM sequences are indicated in red. Exons are indicated by closed boxes and the open boxed areas indicate the restriction sites in each target region. (B) Identification of successfully targeted *Tet1, Tet2*, and *Tet3* genes in mouse haploid ESCs. PCR products were digested with restriction enzymes (BfuAI or MnII) that cleave at the Cas9 endonuclease target sites and then analysed by gel electrophoresis. PCR products generated from clones containing successfully targeted *Tet1*, *Tet2* and *Tet3* genes were uncleaved and were larger than the product generated from the wild-type (WT) clone. The clone numbers are shown above the gel images. The asterisks indicate successfully targeted clones. (C) Sequencing of the successfully targeted *Tet1*, *Tet2* and *Tet3* mutant clones. The PAM sequences are shown in red and the boxed areas indicate the restriction sites in the target regions. Lower case letters indicate insertion mutations and arrows indicate the sites of insertions.

Because haploid cells have only one copy of each chromosome, disruption of one allele can directly cause a loss-of-function phenotype. To examine the efficiency of loss-of-function haploid ESCs, sgRNAs were designed to target the *Tet1*, *Tet2* and *Tet3* genes, which encode members of the tet methylcytosine dioxygenase family (Fig. 1A). Tet proteins convert 5-methylcytosine to 5-hydroxymethylcytosine (5hmC) and this process is an important part of DNA demethylation (Tahiliani et al., 2009). A previous study of the specificity of type II CRISPR suggested that the DNA target site must perfectly match the PAM sequence (NGG) and the 12 bp seed sequence at the 3′ end of the sgRNA (Jinek et al., 2012). The importance of the remaining bases is less well understood and may depend on the binding strength of the matching sgRNA or the inherent tolerance of Cas9 itself. Therefore, we selected a 23-mer sequence (N21GG) from the target gene and used 16 bp of this sequence (N14GG) to search for homologous mouse genes. Sequences that did not cross-react with any other sites in the mouse genome were selected and were used to construct the sgRNA expression vectors. To obtain high expression levels of Cas9 in ESCs, the expression vector was engineered to contain mammalian codon-optimized Cas9 under the control of a CAG promoter (Mali et al., 2013). To examine the efficiency of knockout of each gene, haploid ESCs were co-transfected with the Cas9 expression vector and a sgRNA vector targeting *Tet1, Tet2* or*Tet3*, along with a puromycin marker. Transfected cells were treated with 1 µg/ml puromycin for 48 h. Each targeted locus contains a restriction site; therefore, the cleavage efficiencies of the *Tet1*, *Tet2* and *Tet3* genes were determined by digesting the PCR products with BfuAI (*Tet1* and *Tet3*) or MnII (*Tet2*) (Fig. 1A). Successful targeting was indicated by a disruption of the restriction site; successfully targeted alleles were uncleaved and wild-type alleles underwent complete cleavage. A total of 20 haploid ESC clones from each targeting experiment were screened; the percentages of clones that were successfully targeted were 20%, 30% and 65% for *Tet1*, *Tet2* and *Tet3*, respectively (Fig. 1B). The PCR products of the successfully targeted regions were subcloned and sequenced (Fig. 1C). The results confirmed that each clone contained a mutation in the specific *Tet* gene at the BfuAI or MnII restriction site.

### Simultaneous disruption of the *Tet1*, *Tet2* and *Tet3* genes

**Figure 2:**
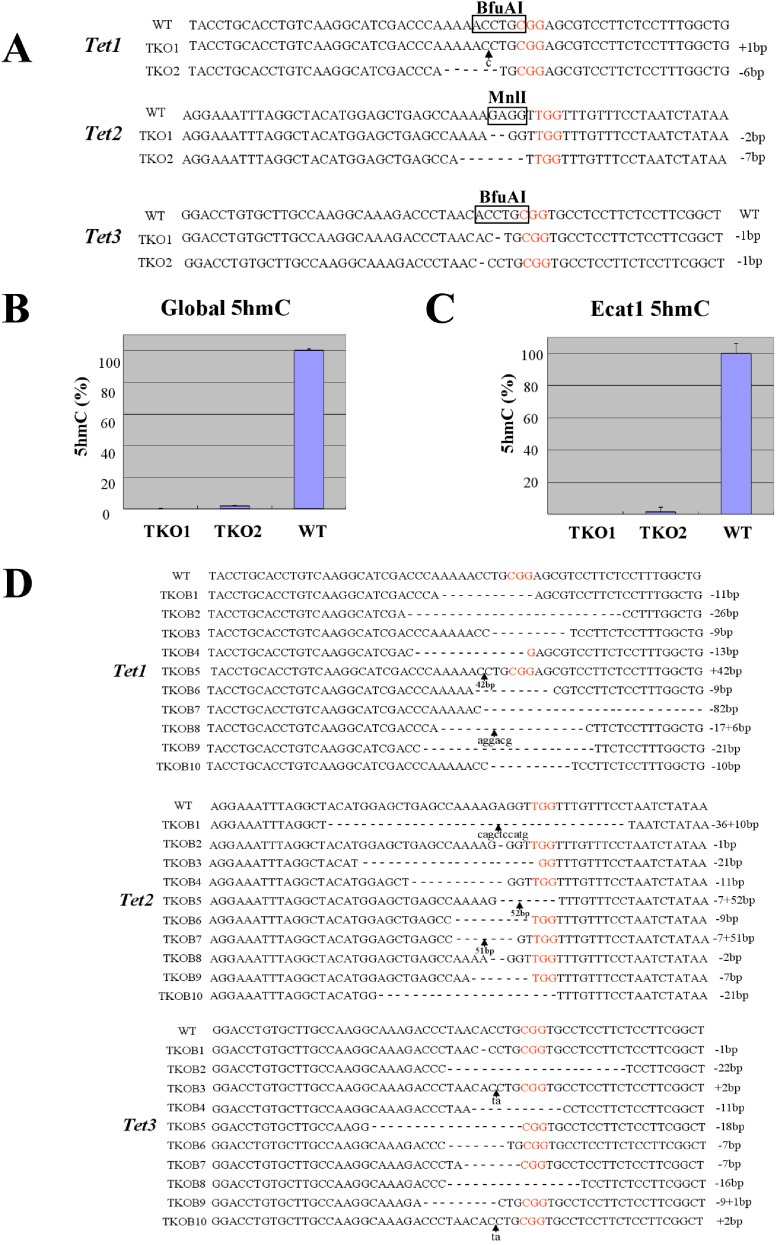
Simultaneous targeting of the *Tet1, Tet2* and *Tet3* genes in mouse haploid ESCs. (A) Sequencing of *Tet1*, *Tet2* and *Tet3* triple knockout mutant clones. The PAM sequences are shown in red and the boxed areas indicate the restriction sites in the target regions. Lower case letters indicate insertion mutations and arrows indicate the sites of insertions. (B, C) Quantification of the 5hmC content in triple knockout clones. Analysis of the global 5hmC levels (B) and the 5hmC levels in the *Ecat1* gene (C) in DNA from two triple knockout (TKO) haploid ESC clones. The quantification of genomic 5hmC was based on the specific transfer of radiolabeled glucose to 5hmC by a purified glucosyltransferase. Data are represented as the mean + SD of *n* = 3 replicate measurements and are shown as a percentage of the 5hmC levels in the wild type (WT). (D) Sequencing of *Tet1*, *Tet2,* and *Tet3* triple knockout mutant clones was obtained following treatment with a high concentration of puromycin. PAM sequences are shown in red and the boxed areas indicate the restriction sites of the target regions. Lower case letters indicate insertion mutations and arrows indicate the sites of insertions.

Higher organisms usually have redundant genes and in these cases it is important to disrupt all members of the gene family simultaneously. Therefore, experiments were performed to determine whether the *Tet1*, *Tet2* and *Tet3* genes could be simultaneously targeted. Haploid ESCs were co-transfected with the Cas9 expression vector and the sgRNA vectors targeting *Tet1*, *Tet2* and *Tet3*, along with a puromycin marker. Transfected cells were treated with 1 µg/ml puromycin for 48 h. Of the 20 haploid ESC clones screened, 2 (10%) were identified as complete triple knockout clones. Sequencing of the subcloned PCR products confirmed that the two triple knockout clones did contain mutations in the *Tet1*, *Tet2* and *Tet3* alleles (Fig. 2A). To determine whether the triple knockout mutants had lost methylcytosine dioxygenase activity, the 5hmC content of the targeted clones was compared to that of wild-type haploid ESCs. Both the global 5hmC content (Fig. 2B) and the 5hmC content of the *Ecat1* gene (Fig. 2C) were markedly lower in the two clones carrying triple mutations than in a wild-type clone. To enhance the efficiency with which triple knockout clones were generated, the concentration of puromycin that cells were treated with was increased to 2 µg/ml. This markedly increased the efficiency with which triple knockout clones were generated to 50%. Of the 20 haploid ESC clones screened, 10 were identified as triple knockout clones (Fig. 2D). Further analysis of two triple knockout ESCs reveals that these cells were become diploid after several passages (Fig. S2). Expression level of pluripotent marker genes (*Oct3/4* and *Nanog*) are not different between triple knockout ESCs and wild type ESCs. However, differentiation markers, such as *Cdx2* (trophectoderm) and Brachyury (mesoderm) were upregulated, whereas *Gata6* (primitive endoderm) were downregulated in triple knockout ESCs (Fig. S3). In addition, these triple knockout ESCs grow more slowly than wild type ESCs (Fig. S4).

### Chromosomal deletions and inversions can be induced by Cas9/RNA-mediated genomic engineering

**Figure 3:**
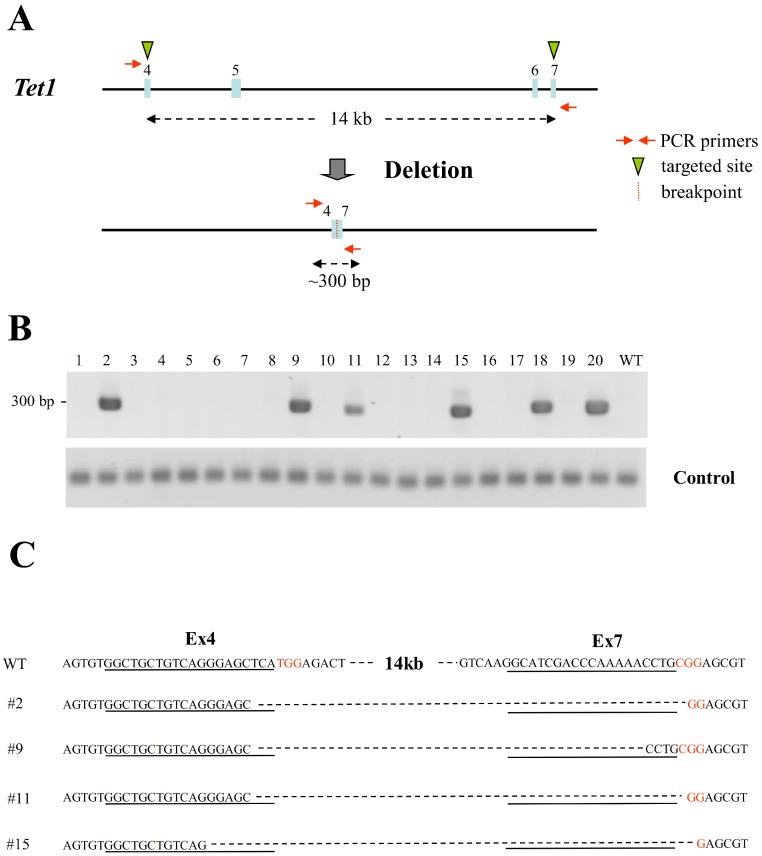
Large chromosomal deletions mediated by two sgRNAs targeting the same chromosome in mouse haploid ESCs. (A) The two sgRNA-targeting sites in the *Tet1* gene. Exons are indicated by closed boxes, the sgRNA-targeting sequences are indicated by green arrowheads, and the PCR primers used for detection of the deletion are indicated by red arrows. (B) Detection of deletions in the *Tet1* gene in ESCs targeted with two sgRNAs. PCRs were performed using primers flanking the sgRNA target sites in exons 4 and 7, as shown in (A). A deletion event resulted in the production of a ∼300 bp product. The clone numbers are shown above the gel image. Control PCR using primers amplifying the *Tet1* region which is not deleted in this experiment was also performed. (C) Sequencing of the PCR products of ESC clones containing *Tet1* deletions. The sgRNA-targeting sequences are underlined and the PAM sequences are indicated in red.

Structural modifications in the form of chromosomal deletions and variable copy numbers account for a significant portion of human genetic variation (Feuk, Carson & Scherer, 2006). Therefore, we investigated whether the simultaneous delivery of two sgRNAs targeting the same chromosome could induce large chromosomal deletions or inversions. Two sgRNAs targeting exon 4 and exon 7 of *Tet1* were used; the distance between the two target sites was 14 kb (Fig. 3A). PCR primers flanking the target regions were used to determine whether the sgRNAs had successfully targeted the DNA. Deletion of the 14 kb sequence was indicated by the generation of a PCR product of approximately 300 bp. A large population (30%) of the 20 transfectant ESCs screened produced the ∼300 bp PCR product indicative of a deletion event (Fig. 3B). Sequencing of these PCR products confirmed large deletions accompanied by the deletion of very few nucleotides at their junctions (Fig. 3C). These results indicate that co-transfection of ESCs with vectors expressing Cas9 and sgRNAs targeting exons 4 and 7 of *Tet1* creates a large chromosomal deletion (14 kb) with high efficiency.

**Figure 4:**
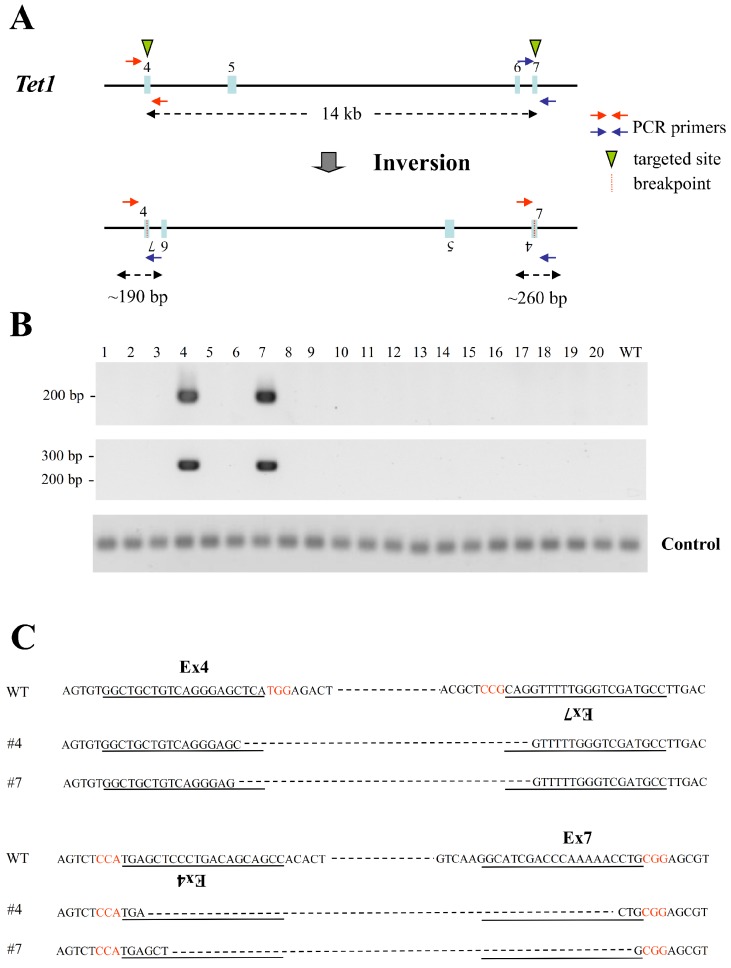
Large chromosomal inversions mediated by two sgRNAs targeting the same chromosome in mouse haploid ESCs. (A) The two sgRNA-targeting sites in the *Tet1* gene. The sgRNA-targeting sequences are indicated by arrowheads and the PCR primer sets flanking the 5′- and 3′-ends of the inversion locus are indicated by red and blue arrows. (B) Detection of inversions in the *Tet1* gene in ESCs targeted with two sgRNAs. PCRs were performed using primers flanking the 5′ and 3′ ends of the inversion locus. An inversion event was indicated by the production of PCR products of approximately 190 bp and 260 bp. The clone numbers are shown above the gel images. Control PCR using primers amplifying the *Tet1* region which is not deleted in this experiment was also performed. (C) Sequencing of the PCR products of ESC clones containing *Tet1* inversions. The sgRNA-targeting sequences are underlined and the PAM sequences are indicated in red. Lower case letters indicate insertion mutations.

The possibility that the two sgRNAs targeting exon 4 and exon 7 of *Tet1* could create inversions was then examined by PCR amplification using primers that spanned the newly created 5′- and 3′-junctions at each exon. Inversion events were indicated by the generation of ∼190 bp and ∼260 bp PCR products (Fig. 4A). Of the 20 transfectant ESCs screened, 10% underwent inversions (Fig. 4B). Sequencing of the two PCR products confirmed inversion events with the deletion of very few nucleotides at their junctions (Fig. 4C). These results indicate that co-transfection of ESCs with vectors expressing Cas9 and sgRNAs targeting exons 4 and 7 of *Tet1* creates a large chromosomal inversion with high efficiency.

## Discussion

Homozygous mutant mammalian cells are useful for studies of gene function; however, the production of homozygous knockouts is time consuming and complicated because diploid cells require disruption of two alleles. The recent development of mouse haploid ESCs (Elling et al., 2011; Leeb & Wutz, 2011) has provided an ideal tool for genetic analyses because haploid cells have only one copy of each chromosome and the disruption of a single allele can directly cause loss-of-function phenotypes. The CRISPR RNA-guided Cas9 nuclease system is a simple and efficient technology for gene targeting (Wiedenheft, Sternberg & Doudna, 2012). Here, we combined the use of haploid ESCs and the CRISPR/Cas9 system to develop a method of studying gene function. Co-transfection of vectors expressing the Cas9 nuclease and sgRNAs targeting *Tet1*, *Tet2* and *Tet3* completely disrupted all three genes and caused loss-of-function phenotypes at high efficiency (50%). This efficiency is remarkably higher than that reported in a recent study of triple knockout ESCs (Wang et al., 2013).

Structural variations in the form of chromosomal deletions, inversions, and changes in copy number account for a significant portion of human genetic variation (Feuk, Carson & Scherer, 2006). This study demonstrates that the Cas9/CRISPR system can be used to generate large chromosomal deletions and inversions in mammals efficiently by a single co-transfection of ESCs with two sgRNAs that target the same chromosome. One of the merits of using haploid ESCs for generating deletions and inversions is that the presence of a single chromosome precludes unintended rearrangements between homologous chromosomes, as reported in diploid cells (Clark et al., 2007). The majority of useful rearrangements will likely occur on autosomes; therefore, the use of Cas9/CRISPR genome engineering of haploid ESCs would be of great merit to applications that extend beyond the modelling of human disease. The generation of large chromosomal deletions could be useful for functional analyses of gene clusters. Inversions are resistant to homologous recombination events (Silver, 1993) and could therefore be used to fix alleles, in a manner analogous to the T-complex.

Although off-target mutations were reported to be high in some cancer cell lines manipulated by CRISPR system (Fu et al., 2013; Hsu et al., 2013) recent reports showed that off–target mutations in pluripotent cells and knockout mice are rare. In addition, two or more interspaced mismatches dramatically reduce Cas9 cleavage (Yang et al., 2013). Another recent report showed that guide-RNA:Cas9 specificity extends past a 7- to 12-base-pair seed sequence. This suggests off-target mutations could be low (Pattanayak et al., 2013).

In summary, the combination of the CRISPR RNA-guided Cas9 nuclease system and haploid ESCs allows efficient genetic manipulation of the mammalian genome; this technique provides a new tool for genetic analyses of complex biological phenomena and diseases.

## Supplemental Information

10.7717/peerj.230/supp-1 Table S1 Derivation of haploid mouse ES cell lines

10.7717/peerj.230/supp-2 Table S2 Primers and target sequences

10.7717/peerj.230/supp-3 Figure S1 Derivation of haploid ES cells A–B, Flow analysis of DNA after propidium iodide (PI) staining of haploid ES cell line Hap F1-2-14 at passage 5 (p5) (A) and Hap F1-2-14 immediately after sorting at p5 (B). The x axis shows fluorescence intensity.

10.7717/peerj.230/supp-4 Figure S2 Generated Tet TKO ES cells have been diplodized after serial culture Flow analysis of DNA after propidium iodide (PI) staining of TKO ES cell lines derived from Hap F1-2-14. The x axis shows fluorescence intensity.

10.7717/peerj.230/supp-5 Figure S3 mRNA expression of pluripotency marker genes and various cell lineages marker genes Oct3/4 and Nanog, pluripotency marker; Cdx2, trophectoderm marker; Brachyury, mesoderm marker; Gata6, primitive endoderm marker; Nestin, neural stem cell marker. Quantitative real-time RT-PCR was performed as previously reported (Horii et al., Cell Reprogram. 12: 551–563, 2010).

10.7717/peerj.230/supp-6 Figure S4 Tet TKO impairs cell proliferation 30,000 cells of TKO1 and WT ES cell lines were seeded onto the 24-well plate, and growth curve were determined by counting the cell numbers every day. Error bars indicate s.d. of three independent experiments.
