# Supplementary material for: Genome engineering of mammalian haploid embryonic stem cells using the Cas9/RNA system
Source: PeerJ. 2013 Dec 23;1:e230. doi: 10.7717/peerj.230 (PMC3883491; doi:10.7717/peerj.230)
Supplement: Table S1 [file peerj-01-230-s001.pdf]

Table S1 Derivation of haploid mouse ES cell lines

Names of ES cell lines	Genetic background	Number of Oocytes activated	Number of 2-cell embryos	Number of morulae	Number of ESC lines obtained	Number of ESC lines with haploid DNA
Hap F1-2-1 to -2-17	B6DBAF1	58	51	26	17	14
Hap B6-1 to -10	B6-EGFP	70	54	17	10	7
