# Supplementary material for: Genome engineering of mammalian haploid embryonic stem cells using the Cas9/RNA system
Source: PeerJ. 2013 Dec 23;1:e230. doi: 10.7717/peerj.230 (PMC3883491; doi:10.7717/peerj.230)
Supplement: Table S2 [file peerj-01-230-s002.pdf]

Table S2 Primers and target sequences

Targets for disruption of genes

Gene Target	Name	Target Sequence
Tet1	gRNA Tet1Ex7	GGCATCGACCCAAAAACCTGCGG
Tet2	gRNA Tet2_9	GGAGCTGAGCCAAAAGAGGTTGG
Tet3	gRNA Tet3Ex7_2	GGCAAAGACCCTAACACCTGCGG

PCR primers for amplification of disrupted genes

Gene Target	Name	PCR Primer Sequence
Tet1	Tet1Ex7-1	CCATCTTCCAGGCGTACCT
	Tet1Ex7-2	AGGCACTGCAACAGGGTTAT
Tet2	Tet2Ex7-1	GCCAGAAGCAAGAAACCAAG
	Tet2Ex7-2	TGTTTAGGGGGTTAGCCAGA
Tet3	Tet3Ex7-3	TGCTTGGCCTATGTTATCCA
	Tet3Ex7-4	GCTCCGAGCATATTTGCAG

Targets for the deletion and inversion

Gene Target	Name	Target Sequence
Tet1	gRNA Tet1_2	GGCTGCTGTCAGGGAGCTCATGG
	gRNA Tet1Ex7	GGCATCGACCCAAAAACCTGCGG

PCR primers for detection of deletion

Target	Name	Target Sequence
Tet1 Ex4-Ex7 deletion	Tet1Ex7-2	AGGCACTGCAACAGGGTTAT
	Tet1Ex4-1	AGAACAAAGCCCCTGTGCTA

PCR primers for detection of inversion

Target	Primer	Target Sequence
Tet1 Ex4-Ex7 inversion left	Tet1Ex7-1	CCATCTTCCAGGCGTACCT
	Tet1Ex4-1	AGAACAAAGCCCCTGTGCTA

Target	Primer	Target Sequence
Tet 1Ex4-Ex7 inversion right	Tet1Ex7-2	AGGCACTGCAACAGGGTTAT
	Tet1Ex4-2	ACCACTCCAAGCCCTTTTCT

Target	Primer	Target Sequence
Tet 1 Control	Tet1RF-1	TTAGCTGGAGGGTGATCGTC
	Tet1RF-2	GTGGGCTGACGTGTGAAAA

*The PAM sequences are indicated in red.
