# Supplementary figures and images for: Genome engineering of mammalian haploid embryonic stem cells using the Cas9/RNA system
Source: PeerJ. 2013 Dec 23;1:e230. doi: 10.7717/peerj.230 (PMC3883491; doi:10.7717/peerj.230)

**a**

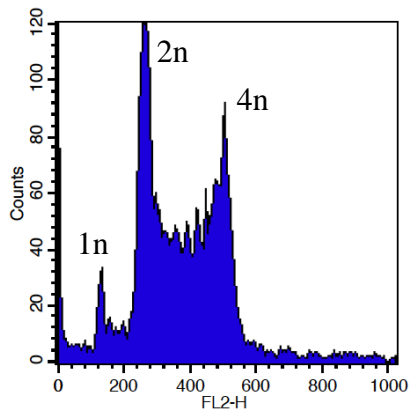

**b**

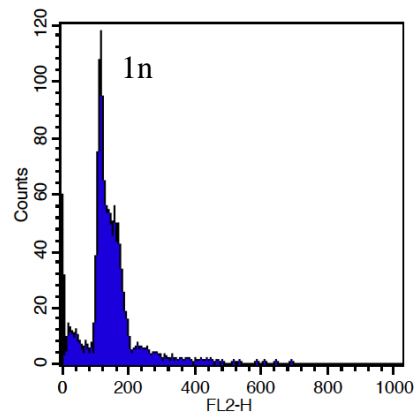

Supplement: Figure S1 — A–B, Flow analysis of DNA after propidium iodide (PI) staining of haploid ES cell line Hap F1-2-14 at passage 5 (p5) (A) and Hap F1-2-14 immediately after sorting at p5 (B). The x axis shows fluorescence intensity. [file peerj-01-230-s003.pdf]

TKO1

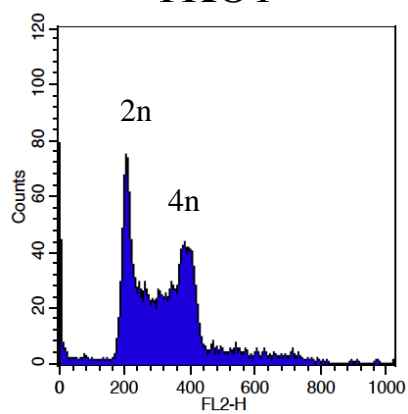

TKO2

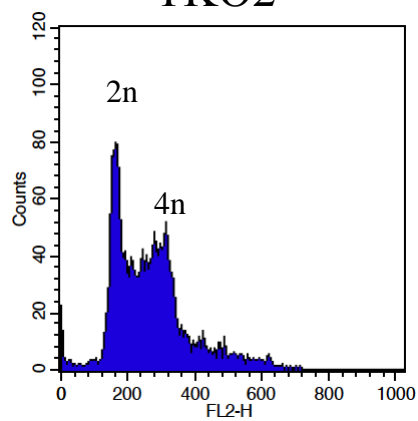

Diploid ES

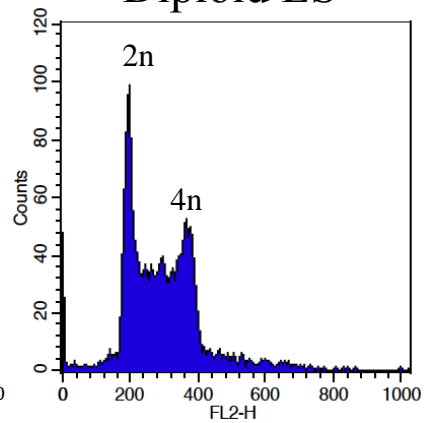

Supplement: Figure S2 — Flow analysis of DNA after propidium iodide (PI) staining of TKO ES cell lines derived from Hap F1-2-14. The x axis shows fluorescence intensity. [file peerj-01-230-s004.pdf]
