# Supplementary material for: Genome engineering of mammalian haploid embryonic stem cells using the Cas9/RNA system
Source: PeerJ. 2013 Dec 23;1:e230. doi: 10.7717/peerj.230 (PMC3883491; doi:10.7717/peerj.230)
Supplement: Figure S3 — Oct3/4 and Nanog, pluripotency marker; Cdx2, trophectoderm marker; Brachyury, mesoderm marker; Gata6, primitive endoderm marker; Nestin, neural stem cell marker. Quantitative real-time RT-PCR was performed as previously reported (Horii et al., Cell Reprogram. 12: 551–563, 2010). [file peerj-01-230-s005.pdf]

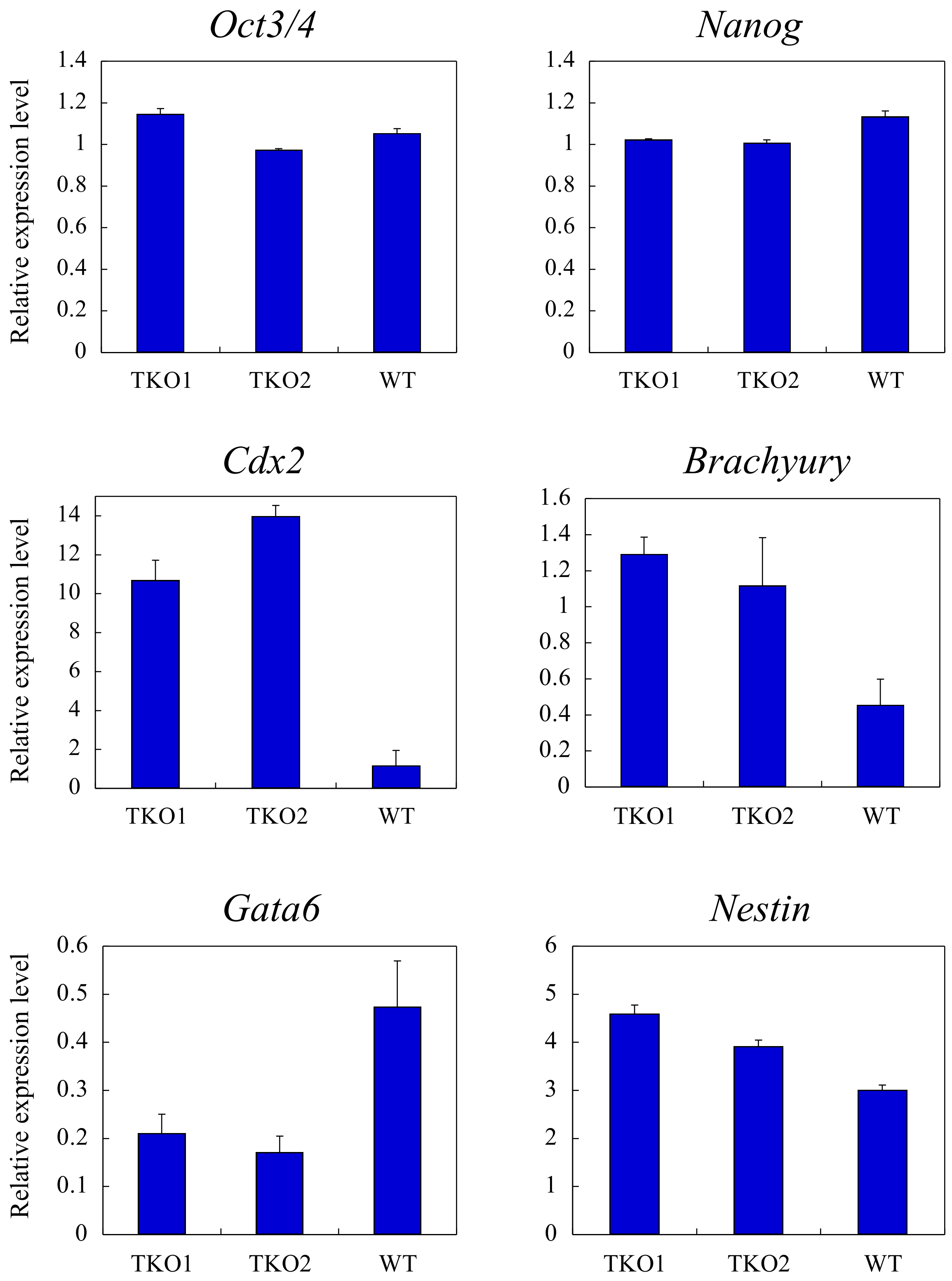

Sup Fig.3 mRNA expression of pluripotency marker genes and various cell lineages marker genes. *Oct3/4* and *Nanog*, pluripotency marker; *Cdx2*, trophectoderm marker; *Brachyury*, mesoderm marker; *Gata6*, primitive endoderm marker; *Nestin*, neural stem cell marker. Quantitative real-time RT-PCR was performed as previously reported (Horii et al., *Cell Reprogram*. 12: 551-563, 2010).
