# Supplementary material for: Genome engineering of mammalian haploid embryonic stem cells using the Cas9/RNA system
Source: PeerJ. 2013 Dec 23;1:e230. doi: 10.7717/peerj.230 (PMC3883491; doi:10.7717/peerj.230)
Supplement: Figure S4 — 30,000 cells of TKO1 and WT ES cell lines were seeded onto the 24-well plate, and growth curve were determined by counting the cell numbers every day. Error bars indicate s.d. of three independent experiments. [file peerj-01-230-s006.pdf]

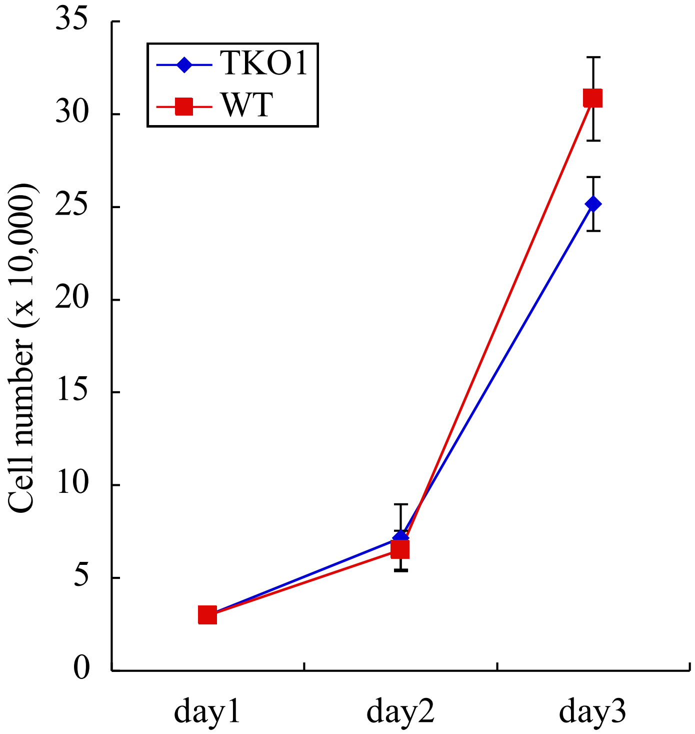

Sup Fig.4 Tet TKO impairs cell proliferation. 30,000 cells of TKO1 and WT ES cell lines were seeded onto the 24-well plate, and growth curve were determined by counting the cell numbers every day. Error bars indicate s.d. of three independent experiments.
